# Prognostic factors for mortality in 123 severe cases of necrotizing fasciitis in 5 hospitals in the Netherlands between 2003 and 2017

**DOI:** 10.1007/s00068-021-01706-z

**Published:** 2021-05-27

**Authors:** Sander van Stigt, Merel Knubben, Tim Schrooten, Edward Tan

**Affiliations:** 1 Department of Surgery, Traumasurgery, Medical Spectrum Twente, Koningsstraat 1, 7512 KZ Enschede, The Netherlands; 2 Department of Surgery, Traumasurgery, Radboud University Medical Center, Nijmegen, The Netherlands

**Keywords:** Necrotizing fasciitis, Prognosis, Mortality, ICU, LRINEC

## Abstract

**Purpose:**

Necrotizing fasciitis (NF) is a severe soft tissue infection with a high morbidity and mortality. With early diagnosis and treatment this could be reduced. Unfortunately, the diagnosis of necrotizing fasciitis can be very difficult. In recent years many risk factors have been identified. In 2004, the Laboratory Risk Indicator for Necrotizing Fasciitis (LRINEC) score was developed. A tool that could help diagnosing NF. In this study, we search for prognostic factors for mortality in necrotizing fasciitis.

**Methods:**

All adult patients with histopathological or surgical confirmed NF needed to be admitted to the intensive care unit for at least 24 h between January 2003 and December 2017 in five hospitals from the Nijmegen teaching region were included. We excluded patients with other forms of soft tissue infections or patients with an intensive care unit (ICU) stay of < 24 h because we exclusively wanted to include patients with a fulminant course of necrotizing fasciitis.

**Results:**

We have included 123 cases. The overall mortality was 31.7% (*N* = 39). The overall mean LRINEC score was 7.4 ± 2.7. Patients who died as the result of NF had a significantly higher median LRINEC score (8 vs. 7, *p* = 0.034). Other parameters found to be associated with mortality are age ≥ 60 years, cardiovascular disease in the medical history, ≥ 2 comorbidities, and lactate level greater than 1.7 mmol/L.

**Conclusion:**

LRINEC score should be calculated in all patients presenting with NF to provide an additional source for clinical outcome. A high LRINEC score could implicate a higher risk of mortality. Especially in elderly patients, with a cardiac history, more than two comorbidities or a lactate level greater than 1.7 mmol/L.

## Background

Necrotizing fasciitis (NF) is a rare but severe soft tissue infection. It is part of the Necrotizing Soft Tissue Infections, and it usually involves the fascia and subcutaneous tissue [1]. The incidence of NF ranges widely. In Thailand, the annual incidence is 15.5 cases per 100,000 [2]. Whereas, it ranges from 0.3 to 5 cases per 100,000 in Western countries [3–6]. This disease was first described by Hippocrates in the fifth century BC [7], but the term necrotizing fasciitis was first used by Wilson in 1952 [8]. Local symptoms of NF include intense pain, erythema, swelling, discolouration of the skin, bullae and subcutaneous emphysema. Systemic symptoms such as fever, nausea, vomiting and general malaise may also occur [9]. Diagnose of NF is difficult, because these symptoms are not specific to NF, but can also be identified in other diseases, like other soft tissue infections (e.g. cellulitis). Symptoms more specific for NF like bullae, necrosis of the skin and subcutaneous emphysema are not commonly seen at initial presentation. Therefore, misdiagnosis is not uncommon (41–96%) [10].

NF could occur spontaneous, without a clear point of entry or after trauma. Anything compromising the integrity of the skin could develop into NF. Examples are surgical wounds, blunt trauma, needle puncture, burns, lacerations and insect bites. Though it’s not rare to find no clear point of entry [9]. Comorbidities which are most commonly associated with NF are diabetes mellitus, smoking/alcohol abuses, liver cirrhosis, HIV infection, malignancy, corticosteroid therapy and chronic renal failure [9, 10].

Depending on the causative organisms, necrotizing fasciitis can be classified into four clinical forms [11]. Type 1 is classified by a synergistic mixture of at least one anaerobic species with one or more facultative anaerobic streptococci and members of the Enterobacteriaceae. Type 2 is usually mono-microbial and caused by group A β-haemolytic streptococcus. Sometimes it is caused by another species of the β-haemolytic streptococcus family or there could be a co-infection with *Staphylococcus aureus*. Type 3 is the effect of an infection with a gram-negative, often marine-related organism. It can be caused by ingestion of seafood or contamination of a wound by water contact. This type is rarely seen in Europe [9, 11]. Type 4 NF is very rare and is caused by *Candida* spp. [11]. It affects mainly immunocompromised patients [12]. Type 1 NF is most prevalent in patients presenting with this infection, with a relative incidence up to 70–80% [13].

The diagnosis of NF can be rather difficult but should be considered in patients presenting with rapidly spreading erythema, pain and discolouration [10]. However, the gold standard for diagnosis is operative exploration. Common intra-operative findings are a greyish coloured and swollen fascia, exudate without purulence (also described as dishwater-like fluid) and easy blunt dissection of tissue planes. This can be further supported with fascia biopsy and tissue culture [11].

Predictors of a greater mortality found by previous studies are a white blood cell count greater than 30 × 10^9 L, creatinine level greater than 176.8 µmol/L, heart disease, old age and female sex [14, 15].

In 2004, Wong et al. developed the Laboratory Risk Indicator for Necrotizing Fasciitis (LRINEC) score as a diagnostic tool. This score consists of six routine laboratory tests: C-reactive protein, leukocyte count, haemoglobin level, sodium level, creatinine level and glucose level. (Table 1). A score of 6 or higher was found to have a positive predictive value of 92% and a negative predictive value of 96% [16]. Previous studies have suggested that the LRINEC score could also be an indication for the risk of mortality in patients presenting with necrotizing fasciitis [17].

**Table 1:** The Laboratory Risk Indicator for Necrotizing Fasciitis (LRINEC score)

	Score
C-reactive protein (mg/L)	
< 150	0
≥ 150	4
Leukocyte count (10^9^/L)	
< 15	0
15–25	1
> 25	2
Haemoglobin (mmol/L)	
> 8.4	0
6.8–8.4	1
< 6.8	2
Sodium (mmol/L)	
≥ 135	0
< 135	2
Creatinine (µmol/L)	
≤ 141	0
> 141	2
Glucose (mmol/L)	
≤ 10	0
> 10	1
Maximum total	13

## Methods

### Study design

This study is an expansion to a study conducted in 2014, which was designed as a retrospective cohort study [1]. The aim of our study was to explore the possibility of prognostic values for mortality.

### Patients

All patients diagnosed with NF between January 2003 and December 2017 in the Radboud University Medical Center Nijmegen (RadboudUMC), the Elisabeth Tweesteden Hospital Tilburg (ETZ), the Gelderse Vallei Hospital Ede (GVH), Rijnstate Hospital Arnhem (RH) and Slingeland Hospital Doetinchem (SH) were included. All these hospitals belong to the same teaching region. The data was transferred to an online data manager (the Research manager™, version 5.20.0.3).

Patients with histopathological or surgical confirmed NF needed to be admitted to the intensive care unit for at least 24 h were included. Patients who had other forms of soft tissue infection or an ICU stay of < 24 h were excluded from this study because we exclusively wanted to include patients with a fulminant course of necrotizing fasciitis. Patients were found using each hospital’s specific patient data system by using diagnostic and procedure codes. A non-WMO declaration was received from the medical ethical testing committee (METC). All local feasibility committees of each of the hospitals assessed and gave approval for expansion of the existing database.

### Data collection

The diagnosis of necrotizing fasciitis was confirmed by histopathologic analysis of tissue samples provided during surgery. If no tissue samples were examined the diagnosis was based on surgical findings, consisting of greyish coloured and swollen fascia, exudate without purulence (also described as dishwater-like fluid) and easy blunt dissection of tissue planes.

We classified all cases into Type 1 or Type 2 NF based on microbiological results. Type 1 was identified as being caused by different combinations of aerobic gram-negative rods of the Enterobacteriaceae group, anaerobic bacteria and streptococci other that *Streptococcus pyogenes*. Type 2 NF was identified as caused by haemolytic streptococcus group A (*Streptococcus pyogenes*), or in rare cases by haemolytic group C, G or *Staphylococcus aureus*.

Using the electronic patient charts demographic data about the patients was collected, as well as vital signs (temperature, blood pressure, heart rate and oxygen saturation), clinical symptoms of the affected body part and laboratory results at presentation. Using the laboratory results, taken on admission the LRINEC score was calculated.

Duration of symptoms, operative findings, the amount and timing of surgeries, wound treatment, results of blood and wound cultures, length of stay at the intensive care unit (ICU), the medium care unit (MCU) and total duration of hospitalisation were noted. Finally, the mortality and the amount of days after which patients died were documented.

### Statistical analysis

The patients were stratified into different groups to evaluate which characteristics could implicate a higher mortality. For continuous variables, it was first assessed whether there was a normality using the Shapiro–Wilk test. When there was normality, a Student *t* test was used to compare the means of both groups. If this was not the case the Mann–Whitney *U* test was used to compare the medians. For categorical variables the Pearson’s Chi-square (*χ*^2^) test was used. A 2-tailed *p* < 0.05 was considered significant. IBM® SPSS® statistics version 25 was used for the analysis of our data.

## Results

### Initial assessment

A total of 123 patients were included (32 RadboudUMC, 34 ETZ, 24 GVE, 23 RH, 10 SH). Seventy-five patients were male (61%). The median age was 59 years, with a mean of 57.5 years (range 21–85 years). The characteristics of our study population are summarised in Table 2.

**Table 2:** Baseline characteristics

Age (years)	57.5 ± 14.2 (21–85)
Gender (% male)	61
Co morbidities(#)^1^	2.2 ± 1.8 (0–8)
Medications	5.2 ± 5.4 (0–27)
Duration of symptoms (days)	2.8 ± 2.8 (1–20)
Known cause (%)	74
Affected body part (%)	
Extremity	49.6 (*N = *61)
Thorax	10.6 (*N = *13)
Abdomen	32.5 (*N = *40)
Fournier	19.5 (*N = *24)
Head/neck	4.9 (*N = *6)
Symptoms at initial presentation (%)	
Pain	69.1 (*N = *85)
Erythema	82.1 (*N = *101)
Swelling	86.2 (*N = *106)
Blisters	20.3 (*N = *25)
Ulceration	21.1 (*N = *26)
Crepitation	13.0 (*N = *16)
Loss of sensibility	2.4 (*N = *3)
Pus	13 (*N = *16)
Other	15.4 (*N = *19)
Time from presentation till surgery (hours)	10.5 ± 12.5 (1.5–82.3) *N = *103
Number of surgeries	3.6 ± 2.5 (0–13)
Mortality (%)	31.7 (*N = *39)
Time till death (days)	9.3 ± 18.2 (1–77)
Hospitalization, excl. Deaths (*N* = 84)	
ICU (days)	11.8 (1–142 ± 17.8)
MCU (days)	2.0 (0–24 ± 4.5)
Ward (days)	26.0 (0–84 ± 19.1)
Total (days)	39.8 (2–117 ± 30.3)
Complications (#)	1.1 ± 1.4 (0–6)
Microorganism(s) found (%)	
Group A hemolytic streptococcus	28.5 (*N = *35)
Group B hemolytic streptococcus	0.8 (*N = *1)
Other monoculture	26.8 (*N = *33)
Mix of microorganisms	42.3 (*N = *52)
No positive cultures	1.6 (*N = *2)
Wound therapy (%), excl. deaths (*N* = 84)	
Synthetic skin dressing + SSG	2.4 (*N = *2)
SSG	48.8 (*N = *41)
VAC	59.5 (*N = *50)
Other	32.1 (*N = *27)
LRINEC score	7.4 ± 2.7 (0–13)

Continuous data is presented as mean with standard deviation and range

*ICU* intensive care unit, *MCU* Medium care unit, *SSG* split skin graft, *VAC* vacuum-assisted closure, *LRINEC* Laboratory Risk Indicator for Necrotizing Fasciitis

^1^Number of diagnosis that patient had prior to presentation with NF: obesity, diabetes, cardiovascular history, malignancy, immune compromised, renal insufficiency, alcohol abuse, chronic liver disease, drug use or other

### LRINEC score

The LRINEC score could be calculated on admission in 107 patients (87%). 84 patients (79%) had a LRINEC of ≥ 6, and 61 patients a score ≥ 8 (56%). In 11 cases the CRP was missing and in 5 cases the glucose level. The mean LRINEC score was 7.4 ± 2.7. A Mann–Whitney *U* test revealed a significant difference in the LRINEC-score of deceased patients (Q_1_ = 7.0 | median = 8.0 | Q_3_ = 10.0, *n* = 31) and those who survived NF (Q_1_ = 5.25 | median = 7 | Q_3_ = 9.0, *n* = 76), *p* = 0.035. This means that patients who eventually died as the result of NF had a higher LRINEC score on admittance into the hospital.

### Mortality rates

A Pearson Chi-square (*χ*^2^) test was used to compare mortality rates in several categorical groups. A statistically different mortality rate was found in patients over the age of 60, patients with a history of cardiovascular disease, two or more comorbidities or a lactate level of 1.7 mmol/L or higher. All results are shown in Table 3.

**Table 3:** Mortality rates in various categories

	Deceased		*p* value
	Yes	No	
Torso vs. Extremities			
Central	35.7% (*N = *20)	64.3% (*N = *36)	1.000
Peripheral	34.7% (*N = *17)	65.3% (*N = *32)	
Type 1 vs 2			
Type 1	37% (*N = *30)	63% (*N = *51)	0.067
Type 2	18.4% (*N = *7)	81.6% (*N = *31)	
Surgery < 24 h vs. > 24 h			
< 24 h	30.9% (*N = *39)	69.1% (*N = *65)	0.992
> 24 h	33.3% (*N = *9)	66.7% (*N = *18)	
Surgery < 6 h vs. > 6 h			
< 6 h	28.3% (*N = *17)	71.7% (*N = *43)	1.000
> 6 h	27.9% (*N = *12)	72.1% (*N = *31)	
Age			
< 60	19.4% (*N = *12)	80.6% (*N = *50)	0.006
≥ 60	44.3% (*N = *27)	55.7% (*N = *34)	
Sex			
Male	29.3% (*N = *22)	70.7% (*N = *53)	0.611
Female	35.4% (*N = *17)	64.6% (*N = *31)	
Diabetes			
Yes	36.4% (*N = *12)	63.6% (*N = *21)	0.650
No	30.0% (*N = *27)	70.0% (*N = *63)	
Cardiovascular disease			
Yes	42.9% (*N = *21)	57.1% (*N = *28)	0.049
No	24.3% (*N = *18)	75.7% (*N = *56)	
Comorbidities			
< 2	16.3% (*N = *8)	83.7% (*N = *41)	0.005
≥ 2	41.9% (*N = *31)	58.1% (*N = *43)	
Leucocytes			
< 30	29.6% (*N = *34)	70.4% (*N = *81)	0.123
≥ 30	62.5% (*N = *5)	37.5% (*N = *3)	
Creatinine			
< 176.8	28.4% (*N = *21)	71.6% (*N = *53)	0.437
≥ 176.8	36.7% (*N = *18)	63.3% (*N = *31)	
Lactate			
< 1.7	8.7%	91.3%	0.036
≥ 1.7	(*N = *2) 33.7% (*N = *8)	(*N = *21) 66.3% (*N = *55)	
CRP			
< 150	29.4% (*N = *5)	70.6% (*N = *12)	1.000
> 150	29.2% (*N = *28)	70.8% (*N = *68)	
Type 1 NF			
Mono culture	42.9% (*N = *15)	57.1% (*N = *20)	0.461
Mix of organisms	32.7% (*N = *17)	67.3% (*N = *35)	

## Discussion

Necrotizing fasciitis is a part of the necrotizing soft tissue infections (NSTI), and still has a high rate of morbidity and mortality. Our existing research/database of 58 was expanded with 85 new cases of NF, bringing the total up to 123 patients [1]. This fact strengthens our position as one of the largest European cohorts. Additional large studies about the LRINEC score have been conducted in Asia, Australia, the US and the Middle-East [16, 18]. The LRINEC score could successfully be calculated in 107 patients, resulting in a mean score of 7.37 (0–13, SD 2.7) The LRINEC score could not be calculated in 16 patients because of missing data. In 11 cases this was because of missing CRP levels, most cases dated from the early 2000’s when CRP was not regularly used to monitor infection.

The value of the LRINEC score has been a point of discussion since publication by Wong et al. in 2004. Although exceptions have been published, we believe, like other larger studies, that the LRINEC score could be of value in the diagnosis and prognosis of necrotizing fasciitis [19]. When a LRINEC score of 7.5 or higher is found we suggest that the patient and relatives should be informed that this could point to a more unfavourable outcome. Especially when this is found in combination with other predictors of higher mortality. Because the LRINEC score was not normally distributed it is more challenging to find a right cut-off value. This value was chosen because of the found difference in mean scores when comparing survivors vs non-survivors. Bechar et al. concluded that the LRINEC score could be improved by including clinical symptoms such as pain, pyrexia and the presence of comorbidities. The LRINEC score is based on an Asian population that includes type 3 necrotic fasciitis caused by vibrio spp. No cases of type 3 NF were found in this study. There is a difference in clinical manifestation between different types of NF [11]. Type 3 necrotizing fasciitis is rare in western countries. The difference between Asian and Western populations could argue in favour of a separate LRINEC score calibrated on a European/Western population.

Previous studies suggest that survival is significantly increased among patients taken to surgery within 24 h after admission compared to those of whom surgery was delayed for more than 24 h [10, 20–22]. In our study, a Chi-square test for independence indicated no significant association between time of presentation till surgery and mortality. 104 patients (85%) included in this study were operated within 24 h after presentation at the hospital. More recent research even suggests that surgery within 6 h has an even better outcome [23]. Some additional analysis were performed using these statements, but we did not find a significant difference in mortality with the time between presentation to surgery being 6 h. This may be explained by the quick and easy availability of an emergency department in the Netherlands. Another explanation could be the inclusion criteria of this study, mainly the minimal duration of ICU admittance being > 24 h. This entails that only patients with a fulminant course of NF were included in the data analysis. Patients who had the biggest benefit of early intervention were excluded. This could also explain the slightly higher mortality rate in our patients in comparison to other studies [10]. Recent studies describe mortality rates between 15 and 49%, with an average mortality of 29% [22, 24]. Our data show an overall mortality of 31.7%.

Other studies suggested that a white blood cell count greater than 30 × 10^9L, creatinine level greater than 176.8 µmol/L, and heart disease at hospital admission were predictors of a higher mortality [15]. Our data only partly supports these claims. Our study only supported the previous data found that a history or cardiovascular disease (CVD) was associated with a higher mortality rate. (42.9% vs. 24.3%, *p* = 0.005). Other studies also found that an increased lactate level was associated with a higher mortality. Our data supported these findings. A cut-off point of 1.7 mmol/L was chosen based on daily practise (33.7% vs 8.7%, *p* = 0.036) [25, 26]. However, our data did not implicate a higher mortality in patients with a white blood cell count greater than 30.000 × 10^3 µL (mortality 62.5% vs. 29.6%, *p* = 0.123) or a creatinine level greater than 176.8 µmol/L (36.7% vs. 28.4%, *p = *0.437).

This could be explained by the small amount of patients (groups of 3 and 5 patients) with a white blood cell count greater than 30.000 × 10^3 µL.

An increased age is also associated with a poor outcome in patients presenting with NF. With a cut-off of an age over 60 or 65 years in past studies [6, 15]. Our data supported both these findings, with a mortality of 44.3% in patients ≥ 60 years vs. 19.4% < 60 years (*p *= 0.006) and a mortality of 47.6% in patients ≥ 65 years vs. 23.5% < 65 years (*p* = 0.012).

Misiakos et al. suggested that there was a greater risk of mortality in females [15]. However, like the study conducted by Benjelloun et al., our data did not support these claims [27]. In females, there was a mortality rate of 35.4% vs. 29.3% in males. This was not a significant difference with a *p* value of 0.611.

A research conducted by Leiblein et al. showed high amputation rates in patients with diabetes mellitus (DM). No significance was found for LRINEC score and age [28]. Goh et al. also described DM as a predictor for higher amputation rates [10]. Using Chi-squared and Mann–Whitney *U* we did an additional analysis to find predictors for amputation. We found higher amputation rates in patients who were immunocompromised (*p* = 0.018) and patients with cardiovascular comorbidities (*p* = 0 044). LRINEC score at presentation was significantly higher in patients who later underwent a amputation. Age was not found as a predictor for higher amputation rates. None of the comorbidities was a predictor for the duration of admission.

As previously mentioned, this is to our knowledge one of the largest databases of patients with necrotizing fasciitis in the Netherlands and Western Europe. Other studies conducted in Europe consist of 15–50 cases [6, 29, 30]. This helped us to determine whether results that were found in studies conducted in Asia, Australia and the Middle East were supported by our data.

There are some limitations to our study. Unfortunately, our research was designed as a retrospective cohort study. Regrettably, because of the long period of data collection, there were some missing data. This was both because of missing lab results, as well as incomplete patient files. As mentioned earlier, this study only included patients with NF that were admitted to an ICU for longer than 24 h. Other forms of soft tissue infections were excluded. Therefore, the study population includes patients with an more fulminant course of NF when compared to other studies. This could have implications on mortality, LRINEC score, lab results and other outcomes.

## Conclusion

Clinical presentation of NF is often with non-specific symptoms such as pain, erythema and swelling. More specific symptoms often develop in hours in the course of NF. Knowing mortality is directly related with time from presentation till surgery, we recommend that the LRINEC score should be calculated in all patients who are presented with the suspicion of NF. Providing an extra tool for diagnosis. Based on our research, if there is a score over 7.5 we recommend to prepare and inform the relatives that this could be an indication of an unfavourable outcome. Especially in elderly patients, with a cardiovascular history, two or more comorbidities or a lactate greater than 1.7 mmol/L.

We would also recommend a prospective approach in further research. Because of the rarity of this disease, maybe even a nationwide approach. This could then be used to validate the LRINEC score as a diagnostic and even prognostic tool in patients presenting with a presumed necrotizing fasciitis. A international European study could be the basis of a new calibration of the LRINEC-score based on a western population consisting of mostly type 1 and 2 NF.

## Data Availability

The dataset used and/or analysed are available from the corresponding author on reasonable request.

## Abbreviations

NF: Necrotizing fasciitis
LRINEC: Laboratory Risk Indicator for Necrotizing Fasciitis
ICU: Intensive care unit
RadboudUMC: Radboud Univeristy Medical Center Nijmegen
ETZ: Elisabeth Tweesteden Hospital Tilburg
GVH: Gelderse Vallei Hospital
RH: Rijnstate Hospital Arnhem
SH: Slingelang Hospital Doetinchem
METC: Medical ethical testing committee
MCU: Medium care unit
SSG: Split skin graft
VAC: Vacuum assissted closure
NSTI: Necrotizing soft tissue infections
CVD: Cardiovascular disease
